# Clinical significance of long noncoding RNA MNX1-AS1 in human cancers: a meta-analysis of cohort studies and bioinformatics analysis based on TCGA datasets

**DOI:** 10.1080/21655979.2021.1888596

**Published:** 2021-03-09

**Authors:** Kang Chen, Jian-Xin Gan, Ze-Ping Huang, Jun Liu, Hai-Peng Liu

**Affiliations:** a Department of General Surgery, Lanzhou University Second Hospital, Lanzhou University, Lanzhou, China; b Department of Anesthesiology, The Third Aﬃliated Hospital, Sun Yat-sen University, Guangzhou, China

**Keywords:** MNX1-AS1, cancer, prognosis, meta-analysis

## Abstract

MNX1-AS1 expression has been proposed to be abnormally upregulated in multiple human malignancies and be linked with the survival outcome of patients. However, relevant conclusions were yielded based on the limited samples. Therefore, we herein implemented a meta-analysis of the published cohort studies to further decipher the relationship of MNX1-AS1 level to prognosis and clinicopathological features in various cancers. Additionally, using The Cancer Genome Atlas (TCGA) datasets we carried out a bioinformatics analysis to make a further evaluation on the prognostic value of MNX1-AS1 expression. The results of meta-analysis indicated elevated MNX1-AS1 level closely correlated with poorer overall survival (OS) (HR = 1.97, 95% CI, 1.73–2.24; P < 0.00001), and disease-free survival (DFS) (HR = 2.24, 95% CI, 1.48–3.38; P = 0.0001) in cancers, which was confirmed by the bioinformatics analysis. Besides, it was observed the upregulated MNX1-AS1 level was significantly related to invasion depth, disease stage, tumor metastasis, and differentiation. Collectively, high MNX1-AS1 level correlated with poor survival outcome and aggressive clinicopathological characteristics in various cancers, suggesting that MNX1-AS1 may be applied as a prognostic marker and even a therapeutic target. Nevertheless, more high-quality studies designed with a large sample size should be conducted to further determine the clinical role of MNX1-AS1 in specific cancer types.

## Graphical abstract

**Figure:**
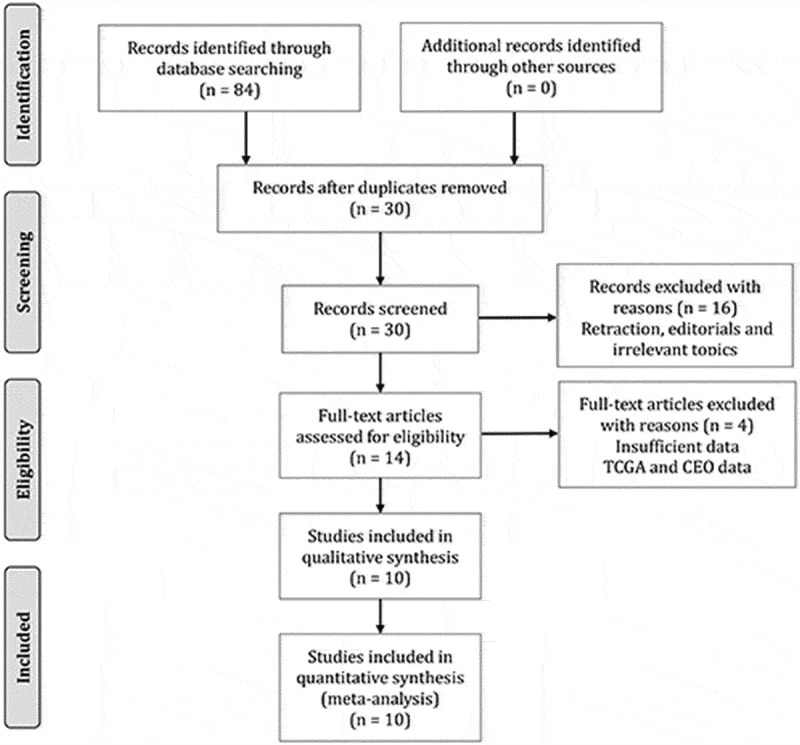

## Introduction

As the incidence and mortality of cancers continuously rise, cancer has become an intractable global public health problem undoubtedly [1]. Although huge advancement in surgical intervention, chemotherapy, and radiotherapy, cancer patients, especially at an advanced stage, still had a much unsatisfactory prognosis [2]. Molecular-targeted therapy has been considered to be a promising therapeutic modality for cancers [3,4]. Therefore, it is very meaningful to identify the molecular biomarkers that correlate with cancer progression and prognosis, so as to provide a theoretical basis for developing individualized treatment target.

Noncoding RNAs such as microRNA, circular RNA, and long noncoding RNA (lncRNA) account for a significant proportion of the human genome [5]. The amount of evidence shows lncRNAs are involved in multiple physio-pathologic processes, such as development, inflammatory response, tissue regeneration, ischemia reperfusion injury, cardiovascular diseases, and cancers [5,6]. In malignancies, numerous lncRNAs are abnormally expressed, which epigenetically and transcriptionally influences expressions of oncogenes and tumor suppressors, suggesting lncRNAs may be developed as the prognostic indicators and treatment targets [7]. For example, long intergenic noncoding RNA01134 was substantially upregulated in hepatocellular carcinoma and it was tightly linked with poor patient outcome [8]. In colon cancer, DNAJC3-AS1 was significantly downregulated, which could promote cancer cell migration, invasion, and epithelial-mesenchymal transition (EMT) through regulating the miR-214-3p/LIVIN [9]. Inversely, LncRNA FLANC level was found to be elevated in colorectal cancer and FLANC could sustain activation of STAT3 signaling by prolonging its phosphorylation, which resulted in VEGFA upregulation to promote angiogenesis, thereby accelerating tumor progression [10]. LncRNA PART1 expression was dramatically reduced in gastric cancer tissues and this dysregulation closely correlated with the unfavorable prognosis of patients who underwent surgery [11]. Further investigation disclosed PART1 exerted a tumor-suppressive effect through upregulating PLZF expression, which recruited EZH2 to induce epigenetic PDGFB silencing, ultimately counteracting PI3K/Akt signaling transduction [11].

MNX1 antisense RNA 1 (MNX1-AS1) was firstly discovered as a highly expressed gene in colorectal malignant tumor, which is termed CCAT5 as well [12]. Recently, a growing number of studies suggested that MNX1-AS1 was aberrantly expressed in diverse malignancies, including cholangiocarcinoma [13], esophagus cancer [14], breast carcinoma [15–17], gastric carcinoma [12,18,19], bladder carcinoma [20], ovarian carcinoma [21,22], cervical cancer [23], osteosarcoma [24,25], laryngeal cancer [26], lung cancer [27–29], prostate cancer [30], hepatocellular carcinoma [15], glioblastoma [31] and colon adenocarcinoma [32]. Moreover, aberrant MNX1-AS1 expression has been proposed to correlated with cancer patient survival, indicating its potential as prognostic marker and therapeutic target [12,15,18,23,26].

Nevertheless, the previous studies were restricted by small sample sizes, in which the clinical significance of MNX1-AS1 level in cancer might be overestimated. Therefore, in this study, we conducted a meta-analysis of the published literature and bioinformatics analysis using TCGA datasets to further evaluate the clinical role of MNX1-AS1 expression in cancers.

## Methods

### Search strategy

‘MNX1-AS1 or CCAT5’ in combination with cancer-related words including ‘cancer,’ ‘tumor,’ ‘carcinoma,’ ‘neoplasm’ ‘adenocarcinoma,’ and ‘malignancy’ were applied to search relevant articles in Chinese National Knowledge Infrastructure (CNKI), PubMed, and Web of Science (WOS) databases from inception to October 2020. Additionally, we also examined the reference lists of the relevant articles to identify eligible studies that could be omitted by electronic search.

### Inclusion and exclusion criteria

Studies that conform to the following items simultaneously were selected [1]: the patients should be definitely diagnosed with cancers by histopathology [2]; the association of MNX1-AS1 with overall survival (OS) or disease-free survival (DFS) was provided regardless of its statistical significance [3]; MNX1-AS1 expression in was detected using polymerase chain reaction (PCR) or in situ hybridization (IHS) [4]; the studies were written in English or Chinese language.

The following conditions were used to exclude the ineligible studies [1]: the articles were not original articles, such as review articles, editorial comments, and meeting abstracts [2]; the studies enrolled the overlapping patient population. In this situation, the latest study was included [3]. The HRs with 95% (CIs) could not be obtained, although the association of MNX1-AS1 expression with survival outcome in cancer patients was explored [4]; the studies analyzed the association of MNX1-AS1 expression with survival outcome in cancer patients based on RNA sequencing data from The Cancer Genome Atlas (TCGA) and Gene Expression Omnibus (GEO) datasets [5]; the studies focused on the irrelevant topics [6]; the studies were conducted in animal models.

### Data collection and quality evaluation

Two investigators collected data from selected articles independently. Any discordance was eliminated via discussion among all the researchers. The collected data included: the first author, the year of publication, region, cancer type, the number of patients, the sex ratio of patients, the age of patients, detection method, antibody type, cutoff of high MNX1-AS1 expression, the percent of patients at TNM III–IV stage, the number of patients with metastasis, the hazard ratios (HRs) with 95% confidence intervals (CIs) for OS and DFS, as well as the analysis method of assessing the prognostic value of MNX1-AS1. HRs from the multivariate analysis were preferentially chosen. If the included studies did not provide HRs, we tried to apply Engauge Digitizer version 4.1 to calculate HRs with CIs from Kaplan-Meier curves [33]. The methodological quality of selected studies was assessed using Newcastle-Ottawa Scale (NOS) system [34]. In this assessment system, three aspects (subject selection, comparability of the subject, and clinical outcome) are evaluated and a score ranging from 0 to 9 may be given to a study. In this meta-analysis, we defined that the studies with more than the mean score of all studies had a high-quality methodology.

### Bioinformatics analysis of TCGA data

Gene Expression Profiling Interactive Analysis (GEPIA), which is supported by TCGA databases, was used for further evaluating the associations of MNX1-AS1 level with OS and DFS. K-M method and log-rank test were employed to perform survival outcome analysis, and the relevant statistical results such as HRs and P values were displayed by K-M curve as previously described [35].

### Statistical analysis

The Review Manager V 5.3 Software (Cochrane Collaboration, London, U.K.) and STATA V 12.0 Software (Stata, College Station, TX) were used for performing statistics analysis in this study. The pooled HRs with 95% CIs were calculated to analyze the association of MNX1-AS1 expression with survival outcomes, and the association between MNX1-AS1 level and clinicopathological parameters was estimated using the pooled odds ratios (ORs) with 95% CIs. We tested the heterogeneity with the help of Cochran’s Q test and the Higgins I-squared statistic, and P value < 0.05 or I2 > 50% suggested heterogeneity was statistically significant. When significant heterogeneity was tested, we conducted the pooled analysis using the random effects model. On the contrast, the fixed effects model was selected [36]. Sensitivity analysis was performed via sequential deletion of a single included study to test if the overall pooled estimation was stable. Egger’s linear regression test and Begg’s rank test were used to assess publication bias [37–39]. P values in this study were two-sided, and P < 0.05 indicated there was a statistical significance.

## Results

Numerous studies have suggested that increased MNX1-AS1 expression might correlate with unfavorable survival outcome in cancer patients. However, the conclusions in those studies may be limited by small sample sizes, probably overestimating the clinical significance of MNX1-AS1 expression in cancer patients. Hence, herein we performed a meta-analysis of the previous studies and bioinformatics analysis based on TCGA datasets to further evaluate the clinical significance of MNX1-AS1 expression in cancers.

### Study search and selection

**Figure 1.**
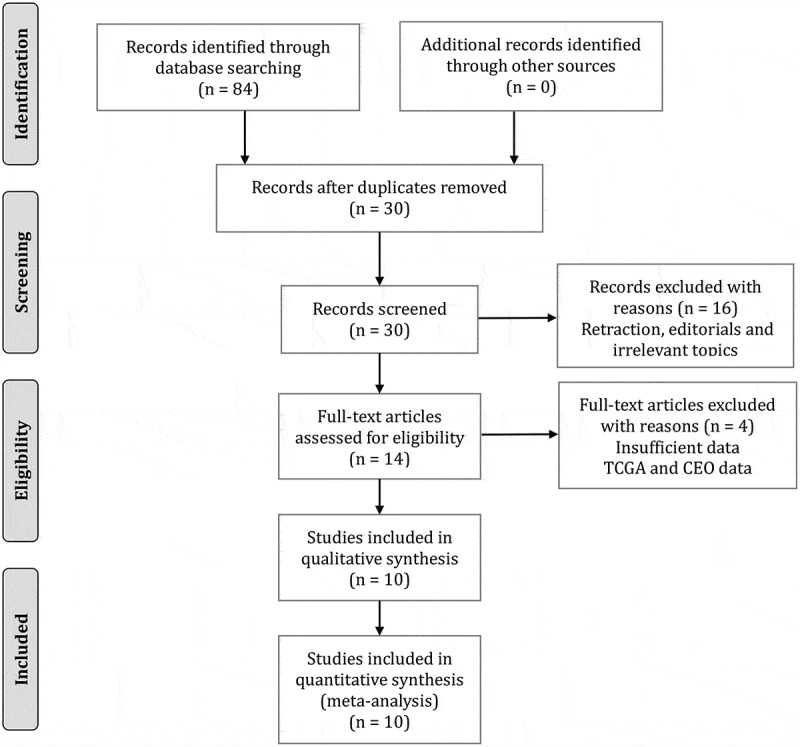
Flow diagram of searching and selecting eligible studies

A total of 84 relevant articles were retrieved from the initial search of PubMed, WOS, and CNKI databases. First, 54 repetitive records were removed. Then, we checked the titles and abstracts of the remained articles and further excluded 16 records for retracted papers, editorials, and irrelevant topics. Next, we carefully reviewed the rest of the articles by full text. In this step, we deleted four articles since they provided unavailable data [40] or data from TCGA or CEO [12,26,27]. Finally, 10 articles were chosen for this meta-analysis [12,15,17–19,22,23,27–29]. The flow diagram of study search and selection is summarized in Figure 1.

### Main characteristics of selected studies

The main characteristics of selected studies are shown in Table 1. All the included studies were published in recent five years and designed with sample sizes from 43 to 177. MNX1-AS1 level was detected through qRT-PCR in nine studies, and by ISH in one study. All these studies were performed in China. Six types of cancer were studied in these studies, including gastric carcinoma, lung carcinoma, hepatocellular carcinoma, breast cancer, cervical cancer, and ovarian carcinoma. Association of MNX1-AS1 level with OS was evaluated in 10 studies, and its prognostic value for DFS was reported in two studies. HRs with CIs were produced by multivariate analysis in five included studies, whereas the other studies only provided survival curves and thereby we calculated HRs with CIs using Engauge Digitizer version 4.1 (Supplement 1). NOS scores of the included studies ranged from 6 to 8.

**Table 1.** The main characteristics of included cohort studies

Study	Year	Country	Cancer type	Sample size	Detection method	Cutoff value	Survival outcome	Hazard ratios (95% CI)	Analysis type	NOS score
Zhang, W	2019	China	Gastric cancer	96	qRT-PCR	Median expression	OS	2.375 (1.839–3.236)	Multivariate	7
Ma, JX	2019	China	Gastric cancer	52	qRT-PCR	Median expression	OS	1.43 (1.06–1.94)	Survival curve	6
Shuai, Y	2020	China	Gastric cancer	174	qRT-PCR	Median expression	OS DFS	2.5 (1.370–4.61) 2.26 (1.317–3.89)	Multivariate	7
Li, AH	2017	China	Ovarian cancer	177	qRT-PCR	Median expression	OS	2.62 (1.193–4.228)	Multivariate	6
Liu, X	2018	China	Cervical cancer	54	qRT-PCR	Mean expression	OS	2.53 (1.07–5.97)	Survival curve	6
Li, JH	2020	China	Breast cancer	95	ISH	Median score	OS DFS	2.261 (1.017–5.027) 2.215 (1.171–4.190)	Multivariate	6
Yang, RH	2018	China	Lung cancer	124	qRT-PCR	Mean expression	OS	2.578 (1.687–3.941)	Multivariate	7
Liu, HB	2018	China	Lung cancer	43	qRT-PCR	Mean expression	OS	2.41 (1.03–5.65)	Survival curve	6
Liu, GF	2019	China	Lung cancer	116	qRT-PCR	Median expression	OS	2.68 (1.49–4.82)	Survival curve	6
Ji, DG	2019	China	Hepatocellular carcinoma	81	qRT-PCR	Median expression	OS	1.55 (1.20–2.01)	Survival curve	6

### Association of MNX1-AS1 level with survival outcome

**Figure 2.**
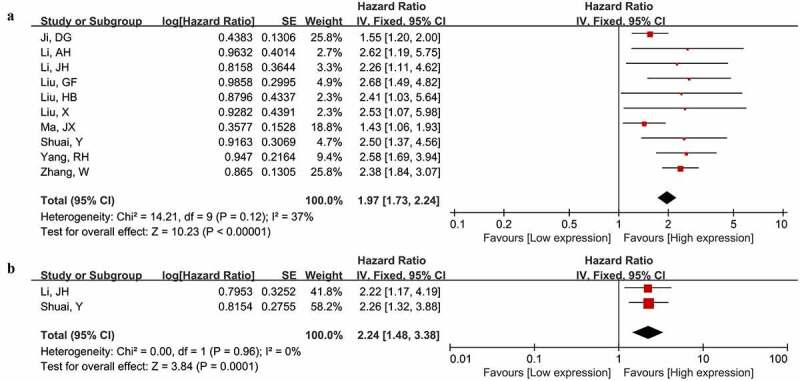
Forest plot illustrated the prognostic significance of MNX1-AS1 level for overall survival (a) and disease-free survival (b)

Ten studies comprising 1119 cancer patients investigate the link between MNX1-AS1 level and OS. Because no significant heterogeneity was identified (I^2^ = 37%, P = 0.12), we conducted the pooled analysis using fixed effects model. The merged HR implied that high MNX1-AS1 level was closely connected with worse OS (HR = 1.97, 95% CI, 1.73–2.24; P < 0.00001) (Figure 2a). Besides, only two studies with 269 patients were included to assess the correlation of MNX1-AS1 expression with DFS. Consistent with OS, it was found increased MNX1-AS1 expression was linked with unfavorable DFS (HR = 2.24, 95% CI: 1.48–3.38; P = 0.0001) (Figure 2b).

### Association of MNX1-AS1 expression with clinicopathologic parameters

**Figure 3.**
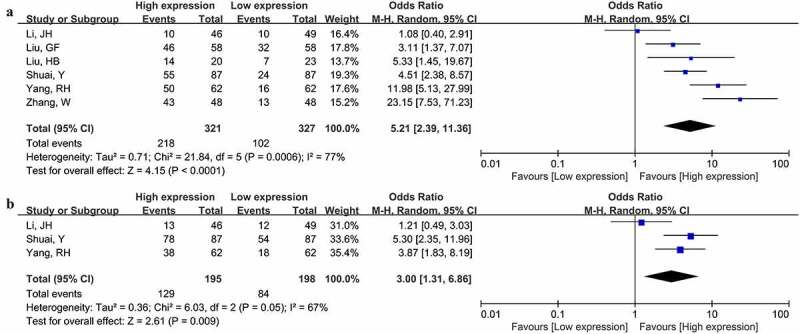
Forest plot illustrated the associations of MNX1-AS1 level with tumor TNM stage (a) and invasion depth (b)

**Figure 4.**
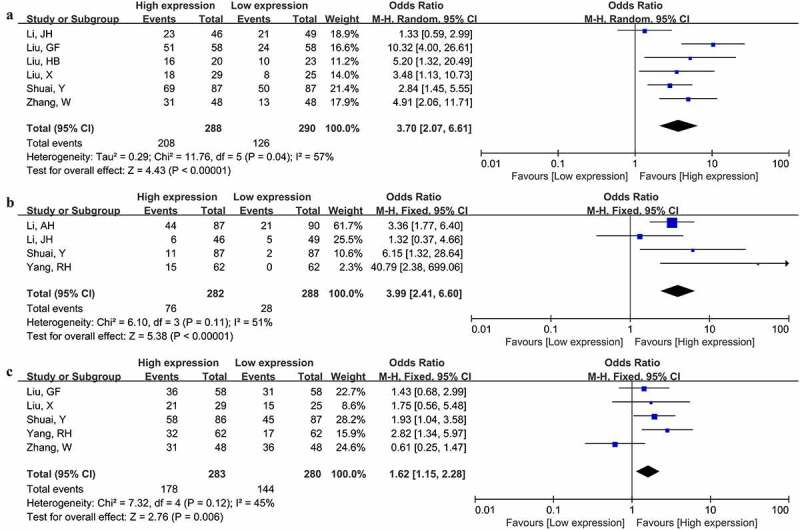
Forest plot illustrated the associations of MNX1-AS1 level with lymphatic metastasis (a), distant metastasis (b) and tumor differentiation (c)

Aggressive clinicopathologic characteristics closely correlate with cancer patient survival. Therefore, we further evaluated the connection of MNX1-AS1 expression with clinicopathologic parameters of cancer patients. As summarized in Table 2, increased MNX1-AS1 level was related to advanced TNM stage (OR = 5.21, 95% CI: 2.39–11.36) (Figure 3a), invasion depth (OR = 3.00, 95% CI: 1.31–6.86) (Figure 3b), lymphatic metastasis (OR = 3.70, 95% CI: 2.07–6.61) (Figure 4a), distant metastasis (OR = 3.99, 95% CI: 2.41–6.60) (Figure 4b) and poor differentiation (OR = 1.62, 95% CI: 1.15–2.28) (Figure 4c). Nevertheless, no significant links of MNX1-AS1 level with age (OR = 1.26, 95% CI: 0.91–1.72) and sex (OR = 1.02, 95% CI: 0.71–1.47) were observed (Table 2).

**Table 2.** The correlation between clinicopathological characteristics and MNX1-AS1 expression

Clinical characteristics	No. of studies	Estimate OR (95%CI)	P value	Heterogeneity	
				I^2^	P value
Age (≤50 vs. >50)	6	1.26(0.91–1.72)	0.16	0%	0.74
Gender (Male vs. Female)	5	1.02(0.71–1.47)	0.89	0%	0.63
Tumor stage (III–IV vs. I–II)	6	5.21(2.39–11.63)	<0.0001	77%	<0.001
Invasion depth (T3-4 vs. T1-2)	3	3.00(1.31–6.86)	=0.009	67%	0.05
Lymphatic metastasis (+ vs. -)	6	3.70(2.07–6.61)	<0.0001	57%	0.04
Distant metastasis (+ vs. -)	4	3.99(2.41–6.60)	<0.0001	51%	0.11
Tumor differentiation (Poor vs. Moderate/Well)	5	1.62(1.15–2.28)	=0.006	45%	0.12

### Sensitivity analysis and publication bias

**Figure 5.**
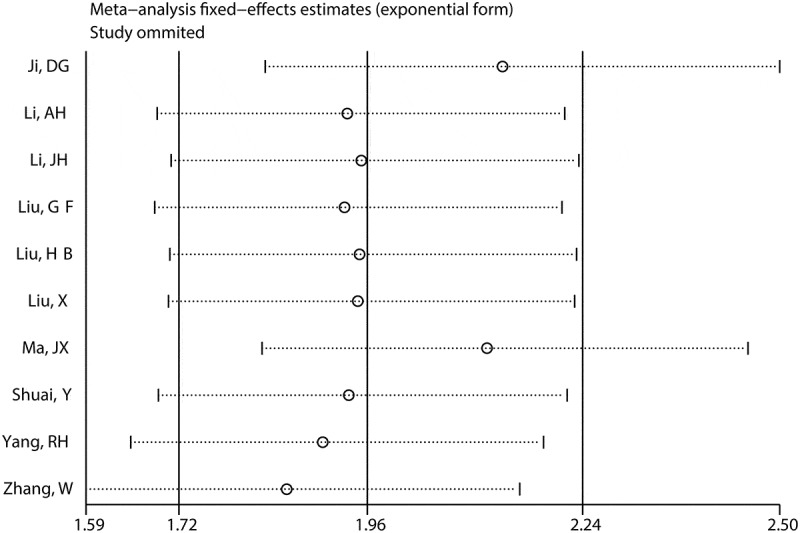
Kaplan-Meier survival curves generated from Gene Expression Profiling Interactive Analysis (GEPIA) illustrated the prognostic significance of MNX1-AS1 level for overall survival (a) and disease-free survival (b) in cancers

Sensitivity analysis was implemented via deletion of a single excluded study in each step. It was observed HRs with CIs for OS did not alter significantly after deleting any eligible study, suggesting the robustness of our combined result (Figure 5). Egger’s linear regression test and Begg’s rank test were used to assess publication bias. The results showed that P values from the two tests were above 0.05 (Supplement 2), indicating there was no significant publication bias. Collectively, these results suggested that our pooled analysis was relatively reliable.

### Validation based on TCGA cohort

Next, we performed bioinformatics analysis using GEPIA to further verify the results of the prognostic value of MNX1-AS1 expression in the meta-analysis above.

**Figure 6.**
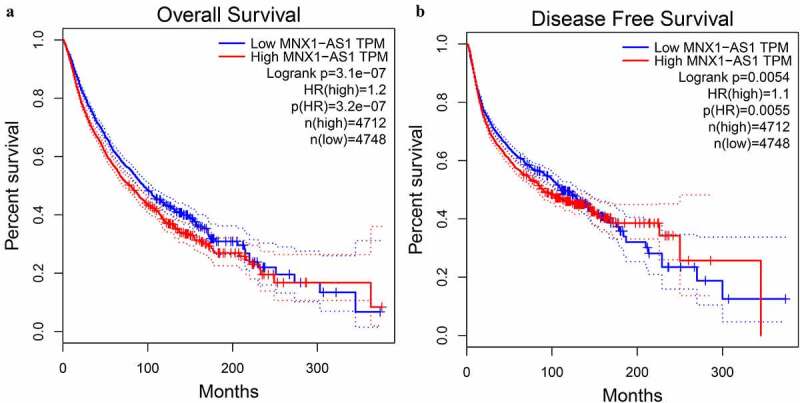
Sensitivity analysis for the pooled estimation of MNX1-AS1 level and OS in cancers

The K-M curves labeled with HRs and P values indicated elevated MNX1-AS1 level was connected with the dismal OS (Figure 6a) and DFS (Figure 6b). Overall, the bioinformatics analysis based on TCGA datasets further validates the findings in the meta-analysis.

## Discussion

Previous studies have reported that MNX1-AS1 level was abnormally upregulated in diverse malignancies, and it was closely related to survival outcome and clinicopathological features of patients. However, the previous studies were restricted by small sample sizes, in which the clinical significance of MNX1-AS1 level in cancer might be overestimated. Therefore, in this study, we implemented a meta-analysis of the published literature and bioinformatics analysis using TCGA datasets to further evaluate the clinical role of MNX1-AS1 expression in cancers. In this meta-analysis, 10 studies comprising 1119 cancer patients were included and the combined results suggested higher MNX1-AS1 level closely correlated with shorter OS and DFS, which was further supported by the bioinformatics analysis. In addition, the combined results showed that increased MNX1-AS1 level was related to multiple aggressive clinicopathologic features, including TNM stage, lymphatic metastasis, distant metastasis, invasion depth, and poor differentiation.

Numerous researchers have tried to decipher the molecular mechanisms accounting for the protumor roles of MNX1-AS1. Lv et al. [21] reported MNX1-AS1 could contribute to ovarian cancer cell proliferation and invasion via upregulation of Bcl-2 family proteins (Bcl-2 and Bax), cyclin-dependent kinase, and cyclin D. Gao et al. [31] demonstrated MNX1-AS1 could enhance the proliferative ability and invasive capacity of glioblastoma cells through targeting miR-4443. In cervical cancer, MNX1-AS1 could promote the proliferation, and suppress the apoptosis of cervical cancer cells via activation of the MAPK signaling pathway [23]. Ye et al. [32] uncovered that MNX1-AS1 could increase colon adenocarcinoma cell growth and metastasis in vitro by regulating the miR-218-5p/SEC61A1 signaling axis. Wang et al. [20] revealed MNX1-AS1 could modulate RAB1A level via inhibiting miR-218-5p to facilitate bladder cancer progression. Similarly, Ji et al. [15] demonstrated that MNX1-AS1 could also target miR-218-5p to increase COMMD8 level in hepatocellular carcinoma cells, consequently accelerating malignant cell growth, motility and invasion in vitro. In lung cancer, MNX1-AS1 was capable of promoting tumor cell proliferation, mobility, and invasion by regulating the miR-527/BRF2 signaling axis [28]. In breast cancer, Cheng et al. [16] first proposed MNX1-AS1 acted as a functional oncogene that could induce the epithelial-mesenchymal transition of cancer cells via activation of AKT/mTOR signaling pathway and upregulation of its natural sense transcript MNX1. Besides, a most recent study revealed MNX1-AS1 could enhance the aggressiveness of triple-negative breast cancer cells through interacting with Stat3 and increasing its phosphorylation in a p-JAK-dependent manner [17]. In prostate cancer, MNX1-AS1 was proved to facilitate cancer cell proliferation, migration, and invasion, but its molecular mechanisms remain unknown [30]. Ma et al. reported MNX1-AS1 was able to foster invasion and metastasis of gastric cancer cells by downregulating CDKN1A [19]. Additionally, Shuai et al. [12] also found upregulation of MNX1-AS1 could promote gastric cancer cell proliferation, motility and invasion and further uncovered that MNX1-AS1 can sponge miR-6785-5p to enhance BCL2 expression in gastric cancer cells [12]. In osteosarcoma, MNX1-AS1 could accelerate osteosarcoma cell proliferation and invasion via downregulating KISS1 [25]. Besides, Wu et al. [24] suggested that MNX1-AS1 could enhance tumor cell epithelial-mesenchymal transition through upregulating MNX1 expression. Autologously, Li et al. uncovered that MNX1-AS1 could also enhance MNX1 transcription, which then activated Ajuba/Hippo signaling pathway in intrahepatic cholangiocarcinoma cells, consequently facilitating tumor progression [13]. In esophagus cancer, MNX1-AS1 was also demonstrated to facilitate the proliferative and invasive capacities of tumor cells through regulating the miR-34a/SIRT1 signaling axis [14]. Collectively, the evidence mentioned above strongly supports that MNX1-AS1 may play a protumor role in human cancers.

Some shortcomings in this meta-analysis should be considered. First, some studies did not report HRs directly. Thus, we calculated HRs manually from Kaplan-Meier survival curves, which may cause operating errors unavoidably. Second, all studies were performed in China. Hence, the results of this meta-analysis may not reflect the clinical significance of MNX1-AS1 expression in other ethnic population. Future studies are needed to determine this issue. Third, the number of studies focusing on a specific cancer type was limited, so the pooled analysis was not conducted in this regard. Obviously, the clinical significance of MNX1-AS1 expression in a specific cancer type should be further validated in more cohort studies. Fourth, this meta-analysis only included studies published in English or Chinese, which might contribute to a bias. Fifth, overall analysis using the GEPIA tool showed that high MNX1-AS1 expression was correlated with worse OS and DFS of cancer patients, whereas the association between MNX1-AS1 and survival outcome was not observed in several cancer types when each type of cancer was analyzed individually. This inconsistency may be partly attributed to the limitation of small sample size, the differences in sequencing technology, and the diversity of tumor genetic backgrounds. Therefore, more studies should be done to further determine the role of MNX1-AS1 in specific cancer types. Sixth, although the current study suggested that MNX1-AS1 may be a potential therapeutic target for cancer, a series of further studies are needed to confirm this point. First of all, more homogeneous clinical studies with larger samples should be conducted to further determine the clinical significance of MNX1-AS1 in each cancer type; In addition, the roles of MNX1-AS1 in cancers and the corresponding mechanisms should be fully elucidated using in vitro cell model and in vivo animal models, such as MNX1-AS1-knockout mice and tumor organoid model; Furthermore, the anticancer effects of MNX1-AS1-targeting drugs should be evaluated in the patient-derived tumor xenograft (PDX) model and tumor organoid model. Next, cautious and stepwise clinical trials may be considered to assess the efficacy of MNX1-AS1-targeting drugs in cancer patients. Seventh, many methodologies are available to evaluate publication bias [39,41–44], but only Egger’s linear regression test and Begg’s rank test were applied in our meta-analysis. Thus, our publication bias evaluation may be not enough reliable, though both Egger’s linear regression test and Begg’s rank test indicated no significant publication bias in the current meta-analysis.

## Conclusion

In summary, high MNX1-AS1 level correlated with poor survival outcome of cancer patients, suggesting that MNX1-AS1 may be exploited as a prognostic marker and even a therapeutic target. However, more high-quality studies designed with a large sample size are demanded to further determine the clinical role of MNX1-AS1 in specific cancer types.

## Supplementary Material

Supplemental Material

## Highlights

Increased MNX1-AS1 level correlated with worse overall survival in cancers

Elevated MNX1-AS1 level correlated with poor disease-free survival in cancers

High MNX1-AS1 expression was related to tumor metastasis
