# Supplementary material for: Clinical significance of long noncoding RNA MNX1-AS1 in human cancers: a meta-analysis of cohort studies and bioinformatics analysis based on TCGA datasets
Source: Bioengineered. 2021 Mar 9;12(1):875–85. doi: 10.1080/21655979.2021.1888596 (PMC8291812; doi:10.1080/21655979.2021.1888596)
Supplement: Supplemental Material [file KBIE_A_1888596_SM5095.zip › KBIE_2020_0181R1_Supplement_1.pdf]

	Ji, DG			Liu, GF			Liu, HB			Liu, X			Ma, JX	
Time(month)	High expression(%)	Low expression(%)	Time(month)	High expression(%)	Low expression(%)	Time(mont	High expres	Low expres	Time(mont	High expres	Low expres	Time(mont	High expres	Low expression(%)
0,206186	99,5918	99,1837	2,01959	99,6226	99,6226	2,41067	99,3243	98,6486	2,15094	98,7387	98,4868	2,27451	99,0099	98,8119
2,26804	99,1837	96,7347	4,13516	96,6038	99,434	4,42409	98,6486	98,6486	4,18868	98,2108	97,9608	3,68627	99,2079	99,4059
3,71134	97,551	97,1429	5,86611	92,4528	99,2453	6,43751	94,5946	98,6486	5,54717	98,6737	97,6922	5,88235	98,8119	98,4158
6,18557	94,6939	94,2857	7,88625	88,4906	98,3019	7,62924	93,9189	94,5946	8,03774	98,142	98,3902	7,92157	98,0198	98,4158
8,24742	94,6939	94,6939	9,90747	83,2075	96,2264	10,0453	94,5946	94,5946	10,1887	98,5993	98,3549	9,96078	94,4554	98,4158
10,1031	92,2449	91,4286	12,0225	81,1321	96,6038	12,4642	94,5946	95,2703	12,2264	98,8233	98,0751	11,8431	91,0891	98,4158
11,9588	91,8367	88,5714	13,66	77,7358	93,5849	13,6695	84,4595	94,5946	14,0377	97,7972	97,5527	14,0392	87,5248	96,0396
14,2268	92,2449	88,9796	15,7779	74,717	90,9434	15,6829	83,7838	94,5946	15,6226	86,6837	97,2804	15,9216	85,3465	96,4356
16,2887	87,7551	88,5714	17,8959	70,9434	88,3019	18,4827	83,7838	89,8649	18,1132	85,915	97,2395	18,1176	81,3861	96,4356
17,9381	85,3061	88,5714	19,7259	63,7736	85,0943	20,0907	72,973	89,1892	19,9245	82,6778	97,7024	20	79,2079	93,2673
19,7938	82,0408	88,9796	22,0352	61,8868	83,3962	22,0824	72,973	83,7838	21,6226	80,672	97,1819	22,1176	75,6436	92,6733
21,8557	79,1837	89,3878	23,8654	58,8679	80	24,0958	60,8108	83,7838	24,1132	77,4347	91,7223	24,0784	72,2772	92,6733
23,9175	75,102	86,5306	25,9831	56,2264	77,5472	26,1092	61,4865	83,7838	26,1509	75,1882	91,4425	26,1176	69,1089	92,6733
25,7732	68,1633	85,7143	28,0998	53,2075	76,2264	28,5226	60,8108	83,1081	27,6226	71,9528	90,9257	28,0784	66,7327	92,2772
28,0412	68,5714	85,7143	29,9267	47,5472	76,2264	30,136	60,8108	83,7838	29,8868	61,5801	92,1201	29,8824	63,3663	88,7129
30,1031	63,6735	85,7143	31,852	44,3396	73,9623	31,7441	60,8108	83,1081	32,6038	54,6445	87,1494	32	58,0198	85,5446
31,9588	64,0816	86,5306	34,2576	43,7736	72,0755	34,1602	60,8108	83,1081	33,9623	53,874	86,6344	33,6471	56,8317	81,3861
34,433	59,1837	81,2245	35,797	41,1321	71,1321	35,7709	60,8108	83,1081	36,3396	50,8886	87,088	35,6863	54,6535	81,7822
36,2887	55,9184	81,2245	37,7204	40	70,7547	37,787	53,3784	83,7838	38,0377	50,8514	84,1045	37,8824	48,3168	70,6931
38,1443	48,1633	81,6327	40,4151	39,8113	68,3019	39,7978	45,2703	83,1081	39,9623	50,5716	67,5704	39,8431	45,3465	70,495
39,7938	48,5714	82,449	42,1477	40,1887	66,4151	41,8112	37,8378	83,1081	42,4528	47,8288	66,298	42,1176	39,0099	70,099
41,6495	48,5714	75,102	44,0704	37,1698	66,7925	44,1946	25,6757	75	43,9245	47,8028	66,5201	44,0784	38,8119	69,901
43,9175	48,5714	69,7959	46,0896	34,717	66,7925	46,2081	26,3514	75	45,8491	46,5396	66,4885	45,8039	39,2079	70,6931
45,9794	48,9796	69,3878	48,3971	33,2075	66,9811	48,2188	26,3514	74,3243	48	47,4951	66,9458	48	38,8119	69,901
47,8351	48,5714	69,7959	49,936	28,8679	66,6038	50,1342	25	50	49,9245	43,267	63,9585	50,0392	39,2079	70,297
50,1031	48,9796	69,3878	52,0532	25,0943	64,717	51,7422	25,6757	49,3243	51,8491	43,2373	62,9417	52	38,6139	70,6931
51,9588	39,5918	68,9796	53,7856	19,8113	63,0189	53,7557	25,6757	49,3243	54,3396	43,2038	62,4082	53,8039	39,4059	70,495
54,0206	31,8367	69,3878	55,9042	17,3585	59,6226	55,7691	25,6757	49,3243	56,3774	37,259	62,8674	55,6078	38,8119	70,297
55,8763	32,2449	68,5714	58,5022	11,6981	57,7358	57,7798	26,3514	48,6486	58,3019	37,7163	62,0968	57,5686	39,0099	69,901
57,9381	32,2449	69,7959	60,0408	11,6981	57,5472	60,1986	24,3243	49,3243	59,8868	36,2088	62,5634	60,0784	39,4059	70,6931
60,2062	32,2449	69,3878												
