# Supplementary figures and images for: Clinical significance of long noncoding RNA MNX1-AS1 in human cancers: a meta-analysis of cohort studies and bioinformatics analysis based on TCGA datasets
Source: Bioengineered. 2021 Mar 9;12(1):875–85. doi: 10.1080/21655979.2021.1888596 (PMC8291812; doi:10.1080/21655979.2021.1888596)

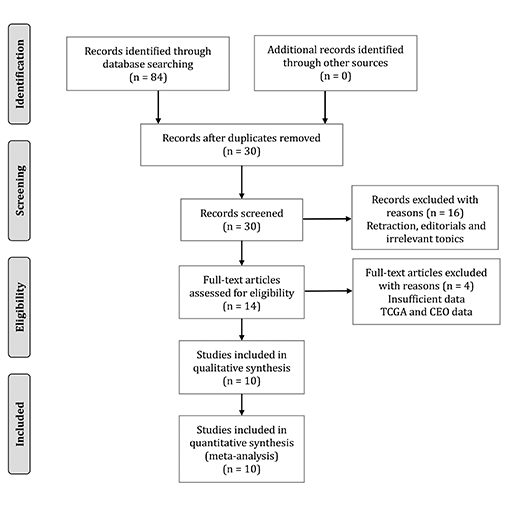

Supplement: Supplemental Material [file KBIE_A_1888596_SM5095.zip › Graph abstract.tif]

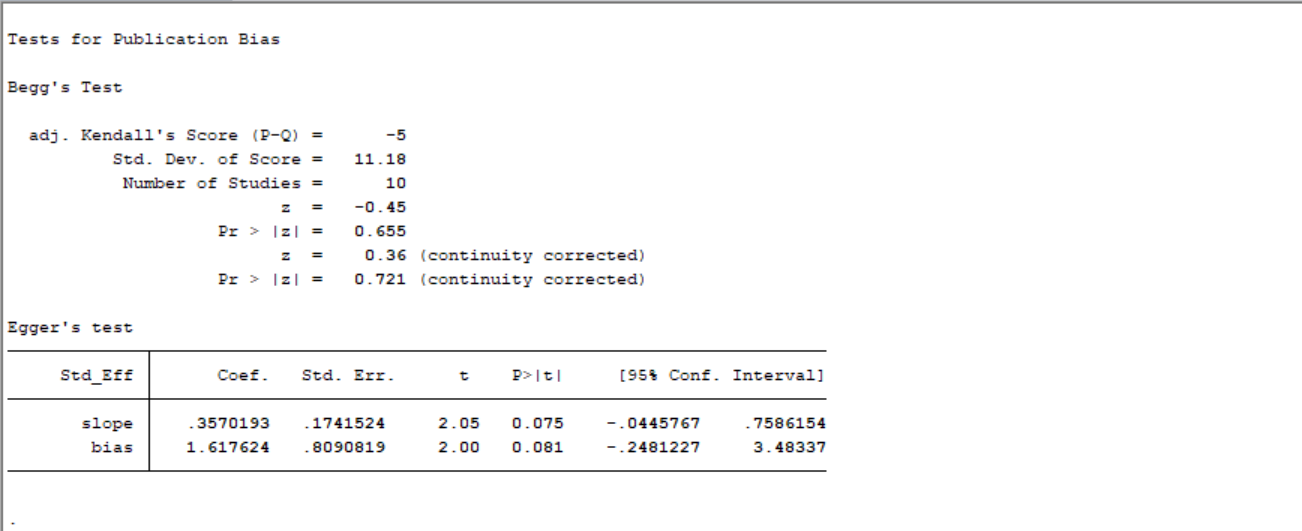

Supplement: Supplemental Material [file KBIE_A_1888596_SM5095.zip › Supplement 2.tif]
